# Assessing performance of single‐sample molecular genetic methods to estimate effective population size: empirical evidence from the endangered *Gochu Asturcelta* pig breed

**DOI:** 10.1002/ece3.2240

**Published:** 2016-06-23

**Authors:** Juan Menéndez, Isabel Álvarez, Iván Fernandez, Nuria A. Menéndez‐Arias, Félix Goyache

**Affiliations:** ^1^ ACGA C/Párroco José Fernández Teral nº 5 A Avilés Asturias 33403 Spain; ^2^ SERIDA‐Deva Camino de Rioseco 1225 Gijón Asturias 33394 Spain

**Keywords:** Coancestry, effective population size, linkage disequilibrium, pedigree, single sample, temporal sampling

## Abstract

Estimating effective population size (*N*_*e*_) using linkage disequilibrium (LD) information (*N*_*e(*_ _*LD*__*)*_) has the operational advantage of using a single sample. However, *N*_*e(*_ _*LD*__*)*_ estimates assume discrete generations and its performance are constrained by demographic issues. However, such concerns have received little empirical attention so far. The pedigree of the endangered Gochu Asturcelta pig breed includes individuals classified into discrete filial generations and individuals with generations overlap. Up to 780 individuals were typed with a set of 17 microsatellites. Performance of *N*_*e(*_ _*LD*__*)*_ was compared with *N*_*e*_ estimates obtained using genealogical information, molecular coancestry (*N*_*e(M)*_) and a temporal (two‐sample) method (*N*_*e(*_ _*JR*__*)*_). Molecular‐based estimates of *N*_*e*_ exceeded those obtained using pedigree data. Estimates of *N*_*e(*_ _*LD*__*)*_ for filial generations F3 and F4 (17.0 and 17.3, respectively) were lower and steadier than those obtained using yearly or biannual samplings. *N*_*e(*_ _*LD*__*)*_ estimated for samples including generations overlap could only be compared with those obtained for the discrete filial generations when sampling span approached a generation interval and demographic correction for bias was applied. Single‐sample *N*_*e(M)*_ estimates were lower than their *N*_*e(*_ _*LD*__*)*_ counterparts. *N*_*e(M)*_ estimates are likely to partially reflect the number of founders rather than population size. In any case, estimates of LD and molecular coancestry tend to covary and, therefore, *N*_*e(M)*_ and *N*_*e(*_ _*LD*__*)*_ can hardly be considered independent. Demographically adjusted estimates of *N*_*e(*_ _*JR*__*)*_ and *N*_*e(*_ _*LD*__*)*_ took comparable values when: (1) the two samples used for the former were separated by one equivalent to discrete generations in the pedigree and (2) sampling span used for the latter approached a generation interval. Overall, the empirical evidence given in this study suggested that the advantage of using single‐sample methods to obtain molecular‐based estimates of *N*_*e*_ is not clear in operational terms. Estimates of *N*_*e*_ obtained using methods based in molecular information should be interpreted with caution.

## Introduction

There is an increasing interest in estimating effective population size (*N*_*e*_) using linkage disequilibrium (LD) information. Although these methodologies have been used basically in natural populations (Waples 1991; Barker 2011), they are of interest in livestock populations with shallow pedigrees in which no sound estimates of effective population size can be obtained using genealogies (Cervantes et al. 2011b).

The advantages of using LD information are clear in terms of time and operational costs: a single sample can provide estimates of, probably, the most important evolutionary parameter for a given population. However, computation of effective population size using linkage disequilibrium (*N*_*e(LD)*_) has well‐known causes of severe bias, namely sample size, markers set size, and minor allele frequency (England et al. 2006; Luikart et al. 2010; Waples and Do 2010).

In any case, the major operational constraint for the estimation of *N*_*e(LD)*_ is that this approach assumes discrete generations and only fit well to semelparous age‐structured species. In iteroparous species, such as livestock, in which generations overlapping is the rule, *N*_*e(LD)*_ values are more likely estimates of the effective number of breeders (*N*_*b*_; effective number of adult individuals that give rise to a cohort) rather than the effective size for a generation (Waples 2006; Barker 2011; Goyache et al. 2011). Although *N*_*e*_ and *N*_*b*_ are closely related, there are large differences between them among species and within populations (Waples et al. 2013, 2014). In such scenario, the analysis of livestock populations with known mating policy, demographic structure, and pedigrees can shed light on the performance of *N*_*e(LD)*_ in natural iteroparous populations.

The demography of a population evolving under generations overlap is likely to affect molecular‐based estimates of *N*_*e*_. When temporal (two‐sample) methods for computing *N*_*e*_ are applied, it is necessary to assume that samples are far from being independent and that “temporal” estimates of *N*_*e*_ must be adjusted using life‐traits data (Jorde and Ryman 1995, 1996). A similar rational has been recently applied to estimates of *N*_*e(LD)*_ obtained using single‐cohort samples. Waples et al. (2014) suggested to adjust estimates of *N*_*e(LD)*_ for demographic bias using the ratio *N*_*b*_/*N*_*e*_. This ratio can be calculated accurately using two key life‐history traits (Waples et al. 2011, 2013).

The demographic concerns described above are not usually addressed even in research carried out in livestock (Corbin et al. 2010, 2012; Flury et al. 2010; Goyache et al. 2011). The endangered Gochu Asturcelta pig breed (Menéndez et al. 2016a) offers a unique scenario to deal with this task. A recovery program for the breed started in 2002 using six founders (three boars and three sows). The reproductive career of the founders and their direct descendants was prolonged as much as possible, and strict breeding policies avoiding matings between close relatives were applied in the population (Menéndez et al. 2016a). This allowed to identify, across years, a number of individuals which could be classified into discrete filial generations: F_1_ (direct descendants of two founders), discrete generation F_2_ (F_1_ × F_1_ crosses),and so on till discrete generation F_5_ (F_4_ × F_4_ crosses). This unique scenario allows to compare, in the same population, estimates of *N*_*e(LD)*_ obtained when discrete generations are considered with those obtained using yearly or biannual cohort samplings. Further, the effect of the correction for demographic bias using parameters obtained via direct observation of the pedigree can also be assessed.

The current research will analyze both the information registered in the herdbook of the Gochu Asturcelta pig breed from 2006 to 2010 and the genotypes obtained for paternity testing. This will allow to assess the performance of *N*_*e(LD)*_ in the following scenarios: (1) samples obtained from discrete filial generations; (2) samples obtained from yearly cohorts; and (3) samples drawn from a number of yearly cohorts equaling to or exceeding generation length. The effect of demographic adjustment of estimates will be assessed as well. For descriptive purposes, performance will be compared with estimates of *N*_*e*_ obtained using single‐sample molecular coancestry, temporal (two‐sample) methods, and genealogical information.

## Materials and Methods

### Data available and sampling

Pedigree data recently analyzed by Menéndez et al. (2016a) were available. Data consisted of 3156 records (including six founders), from 515 litters, with father and mother known, registered in the herdbook of the breeders association (ACGA) from its foundation to August 2014. A total of 109 boars and 309 sows had offspring in data. Genealogies were traced to identify 11 F_1_ individuals (offspring of two founders), 47 F_2_ individuals (F_1_ × F_1_), 216 F_3_ individuals (F_2_ × F_2_), 147 F_4_ individuals (F_3_ × F_3_), and seven F_5_ individuals (F_4_ × F_4_).

Table 1 gives a detailed description of the data used. Analyses were limited to the period in which F_3_ and F_4_ individuals were born (from 2006 to 2010). This ensured that sample size and pedigree depth (at least three equivalents to complete generations; Gutiérrez et al. 2009) were enough to obtain reliable results. Finally, pedigree analyses involved a total of 2248 individuals, born between 2006 and 2010, including 363 F_3_ or F_4_ individuals and 1885 individuals with different pedigree depths due to generations overlap.

**Table 1:** Description of samples used per year of birth. The number of litters and individuals involved in computations are detailed according to pedigree knowledge: (a) those individuals included into discrete generations (F_3_ or F_4_) and (b) those having overlapped genealogies. Both the number of individuals registered in the herdbook (used for genealogical analyses) and the number of individuals typed (in brackets) are given

Year of birth	Discrete generations				Overlapped generations		Totals	
	Litters	F_3_	F_4_	Subtotal	Litters	Individuals	Litters	Individuals
2006	6	39 (32)	0 (0)	39 (32)	5	44 (42)	11	83 (74)
2007	14	99 (98)	6 (6)	105 (104)	18	130 (32)	32	235 (136)
2008	21	50 (34)	109 (95)	159 (129)	51	404 (82)	72	563 (211)
2009	5	22 (21)	26 (26)	48 (47)	85	676 (178)	90	724 (225)
2010	3	6 (6)	6 (6)	12 (12)	79	631 (122)	82	643 (134)
Totals	49	216 (191)	147 (133)	363 (324)	238	1885 (456)	287	2248 (780)

A set of 17 microsatellites (IGF1, S0002, S0026, S0071, S0101, S0155, S0225, S0226, S0227, SW240, SW632, SW857, SW911, SW936, SW951, S0005, and S0090) used in paternity testing and diversity analyses (Menéndez et al. 2015, 2016b) were typed in a representative sample of the available individuals. Most microsatellites used were included in the ISAG–FAO panel (http://www-lgc.toulouse.inra.fr/pig/panel/panel2004.htm). Primer sequences and Polymerase Chain Reaction (PCR) conditions can be found in Menéndez et al. (2016b). PCR was carried out in a GenAmp 9700 thermocycler (Applied Biosystems, Barcelona, Spain) and genotyping was performed on an ABI 3130 DNA‐automated sequencer (Applied Biosystems).

Genotypes of a total of 780 individuals were available. They included: (1) 324 of 363 (89%) F_3_ or F_4_ individuals and (2) 456 of 1885 (24%) individuals with generations overlap in their pedigree born between 2006 and 2010. Altogether, yearly samples available varied from 83 (74‐typed) individuals born in 2006 to 724 (225‐typed) individuals born in 2010 (see Table 1).

According to the structure of data described above, analyses were sequentially carried out on: (1) discrete filial generations (F_3_ and F_4_); (2) yearly cohorts from 2006 to 2010; and (3) sequential biannual samplings mimicking the average generation interval of 1.8 ± 0.03 years reported for the Gochu Astucelta breed by Menéndez et al. (2016a). As mating policy avoids crosses between close relatives (Menéndez et al. 2016a), a model of random mating was assumed when necessary.

### Genealogical estimates of effective population size

The equivalent to complete generations traced (*t*), computed as the sum of (1/2)^*n*^, where *n* is the number of generations separating the individual to each known ancestor (Maignel et al. 1996), was calculated for each individual in the pedigree born in the five‐year period 2006–2010.

Effective population sizes (*N*_*e*_) and their standard errors were estimated on the basis of individual increase in inbreeding Δ*F*_i_ (Gutiérrez et al. 2008, 2009) and coancestry Δ*C*_ij_ (Cervantes et al. 2011a) considering $\Delta F_{i}=1-\sqrt[{t_{i}-1}]{\left(1-F_{i}\right)}$ and $\Delta C_{ij}=1-\sqrt[{(t_{i}+t_{j})/2}]{\left(1-C_{ij}\right)}$ , where *F*_i_ is the inbreeding coefficient of individual *i*,* C*_ij_ is the coancestry coefficient between individuals *i* and *j* (the inbreeding of a descendent from both), and *t*_*i*_ and *t*_*j*_ are their respective equivalent to complete generations. Finally, effective sizes were computed by averaging the individual increase in inbreeding and the increase in pairwise coancestry for all pairs of individuals in a reference subpopulation using the following formulae: $N_{e}F_{i}=1/\left(2\bar{\Delta F_{i}}\right)$ and $N_{e}C_{ij}=1/\left(2\bar{\Delta C_{ij}}\right)$. Finally, following Cervantes et al. (2011a), the ratio *N*_*e*_ *C*_*ij*_/*N*_*e*_ *F*_*i*_ was computed to ascertain the existence of a possible hidden structure in data.

### Single‐sample molecular estimates of effective population size

*N*_*e(LD)*_ was estimated as $N_{e(LD)}=\frac{1}{3({\hat{r}}^{2}-1/S)}$, where ${\hat{r}}^{2}$ is the estimate of the correlation among alleles and *S* is the sample size, using the modification proposed by Waples (2006), which correct for biases resulting from the presence of rare alleles, and was empirically adapted to different sample sizes and mating systems (here large sample sizes, ≥ 30, and random mating apply; see Waples and Do 2010). To check for the consistency of the results obtained, three separate analyses were performed via removing, respectively, alleles with frequencies (*P*_*crit*_) lower than 0.05, 0.02, and 0.01. A jackknife procedure was used to construct 95% confidence intervals of the estimates.

For consistency with the genealogical methods, single‐sample *N*_*e*_ was also estimated using the molecular coancestry method proposed by Nomura (2008) as ${\hat{N}}_{e(M)}=\frac{1}{2{\hat{f}}_{l}}$, where ${\hat{f}}_{l}$ is the average over *n*(*n*−1)/2 pairs of individuals of the molecular coancestry between two individuals *i* and *j* over *L*‐analyzed loci ${\hat{f}}_{ij,l}=\frac{1}{W}\sum {}_{l=1}^{L}w_{l}\frac{f_{ij,l}-{\hat{s}}_{l}}{1-{\hat{s}}_{l}}$, where ${\hat{s}}_{l}$ is the expected homozygosity at a locus *l*. Nomura (2008) followed the suggestion by Oliehoek et al. (2006) of: (1) removing from the computations those alleles alike‐by‐state and not identical‐by‐descent and (2) weighting the contributions over loci using $W=\sum_{l=1}^{L}w_{l}=\sum_{l=1}^{L}\frac{{\left(1-{\hat{s}}_{l}\right)}^{2}}{\sum_{i=1}^{n_{l}}{\hat{p}}_{i}^{2}\left(1-\sum_{i=1}^{n_{l}}{\hat{p}}_{i}^{2}\right)}$ , where *p*_*i*_ is the frequency of the allele *i* at a given locus, to increase the importance of loci with small *s*_*l*_ and balanced allele frequencies. This method uses alleles at any frequency for computations. A jackknife procedure was used to construct 95% confidence intervals of the estimates.

### Two‐sample molecular estimates of effective population size

To illustrate differences between single‐sample and two‐sample estimators of molecular‐based *N*_*e*_, the unbiased temporal method proposed by Jorde and Ryman (2007), which has been proved to give consistent estimates across cohort pairs in a livestock framework (Goyache et al. 2011), was performed as well. This method is based on the computation of the estimator *F*_*s*_, computed as

(1) $$F_{s}=\frac{\sum_{i=1}^{A}{\left(x_{i}-y_{i}\right)}^{2}}{\sum_{i=1}^{A}z_{i}{\left(1-z_{i}\right)}^{2}}$$,

where *A* is the number of alleles at the locus, *x*_*i*_ and *y*_*i*_ are the frequencies of the *i*th allele in the first and second samples, respectively, and *z*_*i*_ is the average frequency of the *i*th allele over samples. Computations were performed under a sampling plan I (Waples 1989) in which individuals are sampled nondestructively and subsequently returned to the population. Under this sampling plan, the Jorde and Ryman's (2007) estimator of *N*_*e*_ (here noted as *N*_*e(JR)*_) is $N_{e(JR)}=\frac{F_{s}\left[1-1/(4\tilde{n})\right]-1/\tilde{n}+1/N}{\left(1+F_{s}/4\right)\left[1-1/(2n_{y})\right]}$, where *n*_*y*_ is the number of individuals in the second sample, $\tilde{n}$ is the harmonic mean of the sample sizes *n*_*x*_ and *n*_*y*_, and *N* is the actual census size of the population at the time of first sampling. A jackknife procedure was used to construct 95% confidence intervals of the estimates.

### Demographic adjustment for bias

Following Waples et al. (2014), *N*_*e(LD)*_ estimates were corrected dividing them with the ratio *N*_*b*_/*N*_*e*_ *,* where *N*_*b*_ and *N*_*e*_ are the effective number of breeders and effective population size, respectively. Ratio *N*_*b*_/*N*_*e*_ was estimated from demographic information using a discrete‐time, age‐structured, and deterministic model and using age‐specific survival rates (*s*_*x*_) and birth rates (*b*_*x*_) calculated separately for males and females (Table S1). The model assumes that: (1) reproduction occurs at intervals of exactly one time unit (here one year); (2) survival and fecundity are independent of events in previous time periods; (3) there is no upper bound to the number of offspring an individual can produce in one breeding cycle; and (4) individuals survive to their first birthday and, therefore, fecundities are scaled to result in a stable population that produces a fixed number (N_1_) of individuals per cohort that survive to age 1.

Following Jorde and Ryman (1995, 1996), *N*_*e(JR)*_ estimates were corrected multiplying them with the ratio *C*/*G,* where *C* is a correction factor obtained from life table data (see Table S2) and *G* is the generation interval. Factor *C* accounts for variance due to mortality as a cohort passes from one‐year class to the next and for genetic covariance among cohorts (because individuals from multiple age classes are the parents of a given cohort). The model to compute factor *C* requires a basic life table with information on age‐specific survival rates (*l*_*i*_) and birth rates at each age class *i* (i.e., gametic contribution; *b*_*i*_; see Table S2).

In all cases, life table data were estimated directly from the Gochu Asturcelta pig pedigree limiting the age of the parents to 5 years old.

### Software used

All demographic and genealogical analyses were computed using the program ENDOG v4.8 (Gutiérrez and Goyache 2005) freely available at http://www.ucm.es/info/prodanim/html/JP_Web.htm

Molecular‐based estimates of *N*_*e*_ were computed in all cases using the program NeEstimator (Do et al. 2014) freely available at http://www.molecularfisherieslaboratory.com.au/neestimator-software/.

Ratio *N*_*b*_/*N*_*e*_ was computed from life table data using the program AgeNe (Waples et al. 2011, 2013) freely available at http://conserver.iugo-cafe.org/user/Robin%20Waples/AgeNe.

The Jorde and Ryman's (1995, 1996) correction factor *C* was computed using a program kindly provided by Dr P. E. Jorde (http://folk.uio.no/ejorde/software/factorc.zip).

## Results

Table 2 gives the estimates of *N*_*e*_ obtained using linkage disequilibrium (*N*_*e(LD)*_), molecular coancestry (*N*_*e(M)*_), and pedigree information. When discrete filial generations were considered, genealogical estimates of *N*_*e*_ were consistently the same varying from *N*_*e*_ *F*_*i*_ = 5.0 ± 0.8 for F_3_ to 5.6 ± 0.3 F_4_. Estimates of *N*_*e*_ *F*_*i*_ and *N*_*e*_ *C*_*ij*_ were comparable across yearly and biannual samplings with the lower estimates for the cohort sampled in 2006. Parameter $\bar{\Delta F_{i}}$ tended to have similar values across either yearly or biannual samplings. However, both *N*_*e*_ *F*_*i*_ and *N*_*e*_ *C*_*ij*_ tended to increase with pedigree depth (and size of the breeding stock) varying from *N*_*e*_ *F*_*i*_ = 4.6 ± 1.9 for Cohort_2006_ to *N*_*e*_ *C*_*ij*_ = 9.2 ± 0.3 for Cohort_2010_. Further, ratio *N*_*e*_ *C*_*ij*_/*N*_*e*_ *F*_*i*_ was roughly 1 for F_3_ and F_4_. However, this ratio increased with years from 1.09 for Cohort_2006_ to 1.39 for Cohort_2010_, therefore suggesting the existence of a slight hidden structure in the Gochu Asturcelta pedigree (Table 2).

**Table 2:** Number of individuals (*N*) involved and estimates of effective size for each discrete generation, yearly cohort, and biannual sampling analyzed in the Gochu Asturcelta pig breed population computed via molecular‐based methods (linkage disequilibrium, *N*_*e(LD)*_ and molecular coancestry, *N*_*e(M)*_) and pedigree information (individual increase in inbreeding, *N*_*e*_ *F*_*i*_ and individual increase in coancestry, *N*_*e*_ *C*_*ij*_). In brackets, confidence intervals of the estimates on 95% (molecular‐based methods) or standard errors of the estimates (genealogical methods) are provided. Additionally, the estimated correlation (${\hat{r}}^{2}$) and molecular coancestry ($\hat{f}$) among alleles are given for the molecular‐based methods and mean inbreeding (*F*), mean equivalent to discrete generations (*t*), and average individual increase in inbreeding ($\bar{\Delta F_{i}}$) are provided for pedigree data

Sampling	Molecular estimates					Genealogical estimates						
	*N* b	${\hat{r}}^{2}$	*N*_*e(LD)*_ c	$\hat{f}$	*N*_*e(M)*_	*N* b	*F*	*t*	$\bar{\Delta F_{i}}$	*N*_*e*_ *F*_*i*_	*N*_*e*_ *C*_*ij*_	*N*_*e*_ *C*_*ij*_ */N*_*e*_ *F*_*i*_
Discrete generations												
Generation 3	191	0.00532	17.0 (14.4; 19.9)	0.0681	7.3 (5.2; 9.9)	216	0.20 ± 0.08	3	0.10 ± 0.04	5.0 ± 0.8	5.4 ± 0.5	1.08
Generation 4	133	0.00770	17.3 (13.5; 21.8)	0.0951	5.3 (2.3; 9.3)	147	0.25 ± 0.04	4	0.09 ± 0.01	5.5 ± 0.2	5.6 ± 0.3	1.02
Yearly sampling												
Cohort_2006_	74	0.01410	6.3^d^ [9.4^e^ (7.7; 11.2)]	0.0925	5.4 (4.1; 6.8)	83	0.17 ± 0.15	2.7 ± 0.7	0.10 ± 0.10	4.6 ± 1.9	5.0 ± 0.6	1.09
Cohort_2007_	136	0.00753	27.5^d^ [41.2^e^ (31.9; 53.7)]	0.0390	12.8 (3.1; 29.4)	235	0.20 ± 0.10	3.2 ± 0.4	0.10 ± 0.05	5.2 ± 1.0	6.2 ± 0.4	1.19
Cohort_2008_	211	0.00481	16.9^d^ [25.3^e^ (20.5; 31.1)]	0.0666	7.5 (4.1; 12)	563	0.22 ± 0.05	3.8 ± 0.4	0.08 ± 0.02	6.0 ± 0.6	7.2 ± 0.4	1.20
Cohort_2009_	225	0.00451	18.9^d^ [28.4^e^ (23.3; 34.4)]	0.0442	11.3 (6.3; 18.8)	724	0.22 ± 0.05	4.2 ± 0.5	0.08 ± 0.02	6.3 ± 0.8	8.3 ± 0.4	1.32
Cohort_2010_	134	0.00764	13.2^d^ [19.8^e^ (16.4; 23.7)]	0.0062	8.4 (0.1; 40.4)	643	0.25 ± 0.07	4.7 ± 0.6	0.08 ± 0.02	6.6 ± 0.7	9.2 ± 0.3	1.39
Biannual sampling^a^												
Sampling_2006–2007_	210	0.00483	20.1^d^ [30.2^e^ (25.8; 38.3)]	0.0500	10.0 (6.3; 14.5)	318	0.19 ± 0.11	3.1 ± 0.5	0.10 ± 0.06	5.0 ± 1.4	5.7 ± 0.5	1.14
Sampling_2007–2008_	347	0.00291	24.2^d^ [36.3^e^ (29.3; 44.6)]	0.0356	14.1 (4.6; 28.8)	798	0.21 ± 0.07	3.6 ± 0.5	0.09 ± 0.03	5.7 ± 0.8	6.7 ± 0.4	1.18
Sampling_2008–2009_	436	0.00231	20.0^d^ [30.0^e^ (25.2; 35.5)]	0.0570	8.8 (5.7; 12.5)	1287	0.22 ± 0.05	4.0 ± 0.5	0.08 ± 0.02	6.2 ± 0.7	7.7 ± 0.4	1.24
Sampling_2009–2010_	359	0.00281	21.8^d^ [32.7^e^ (28.3; 37.7)]	0.0240	20.8 (7.2; 41.5)	1367	0.24 ± 0.06	4.4 ± 0.6	0.08 ± 0.03	6.5 ± 0.7	8.6 ± 0.4	1.32

a Sampling mimicking the mean generation interval reported by Menéndez et al. (2016a) for the whole pedigree of the Gochu Astucelta breed (1.8 ± 0.03 years).

b Number of individuals involved in the estimates.

c Values obtained removing alleles with frequencies (*P*_*crit*_) lower than 0.05.

d Estimates of effective size after correction for bias due to age structure.

e Original estimates of effective size and confidence intervals.

*N*_*e(LD)*_ took values over 17.0 for both discrete filial generations F_3_ and F_4_ (Table 2). Estimates of *N*_*e(LD)*_ obtained for yearly or biannual samplings were adjusted for generations overlap using the ratio *N*_*b*_/*N*_*e*_ computed using demographic information. This ratio took a value of 0.667 corresponding to demographic estimates of *N*_*b*_ and *N*_*e*_ of 222.9 and 334.9, respectively. Estimates of *N*_*e(LD)*_ were highly consistent no matter the *P*_*crit*_ used. Therefore, only estimates obtained using *P*_*crit*_ = 0.05 are given. When yearly samplings were considered, the corrected estimates were similar to those obtained for the discrete filial generations when sample size was high (16.9 for Cohort_2008_ and 18.9 for Cohort_2009_). However, when sample size (Cohorts 2006, 2007, and 2010) was lower, estimates were clearly biased downward or upward. Using biannual samplings, mimicking the average generation interval as recommended by Waples et al. (2013, 2014), the corrected estimates were biased upward varying from 20.0 for Sampling_2008–2009_ to 24.2 for Sampling_2007–2008_. The increase of sampling period to three years did not change the scenario described above (Table S3). In any case, it is worth mentioning that before demographic correction (using ratio *N*_*b*_/*N*_*e*_), *N*_*e(LD)*_ estimates were always unacceptably biased upward (Table 2).

In general, estimates of *N*_*e(M)*_ took lower values than their *N*_*e(LD)*_ counterparts (Table 2). *N*_*e(M)*_ for discrete filial generation F_4_ (5.3) was significantly lower than that of F_3_ (7.3) due to a noticeable increase in molecular coancestry (9.51% vs. 6.81% in F_3_). When yearly or biannual samplings were considered, estimates of *N*_*e(M)*_ followed a similar trend to those of *N*_*e(LD)*_: the higher the *N*_*e(LD)*_ values the higher the *N*_*e(M)*_ estimates. Except for Sampling_2008–2009_, decreases in ${\hat{r}}^{2}$ coincided with lower molecular coancestry values leading to estimates of *N*_*e(M)*_ and *N*_*e(LD)*_ highly biased upward (see Cohort_2007_ in Table 2). Again, the increase of sampling period to three years did not give any improvement in estimating *N*_*e*_ (Table S3).

Estimates of *N*_*e*_ were also obtained using a temporal method, previously tested in the livestock framework (Goyache et al. 2011), to gain more evidence on performance of single‐sample methods to estimate *N*_*e*_ when samples are drawn from a number of yearly cohorts (Table 3). Estimates were corrected for overlapping generations by multiplying the original values with the ratio *C*/*G* (2.23) corresponding to a correction factor, *C*, computed following Jorde and Ryman (1995, 1996), of 4.01. Although the program FactorC gave an estimate of generation interval, *G*, of 1.93 years, the “real” *G* of the population of 1.8 (± 0.03) years reported by Menéndez et al. (2016a) was used to the risk of slightly overestimate the *N*_*e(JR)*_ values. When subsequent yearly samplings were considered, the estimates of *N*_*e(JR)*_ had a noticeable variation fluctuating from 13.4 (Cohort_2006_ − Cohort_2007_) to 33.0 (Cohort_2009_ − Cohort_2010_). When the two samples used were separated by three years, the estimates obtained become more consistent varying from 23.6 (from Cohort_2008_ to Cohort_2010_) to 25.8 (from Cohort_2007_ to Cohort_2009_), therefore suggesting that drift signal was not strong enough in subsequent yearly samplings to give reliable estimates of *N*_*e*_. Note that the estimates of *N*_*e(JR)*_ for three‐year samplings were slightly higher to the adjusted *N*_*e(LD)*_ estimates obtained for biannual samplings (Table 2) and slightly lower to the adjusted and three‐year sampling *N*_*e(LD)*_ estimates (Table S3). In any case, these *N*_*e(JR)*_ and *N*_*e(LD)*_ estimates were fully comparable.

**Table 3:** Estimates of *N*_*e*_ obtained in the Gochu Asturcelta pig population using the temporal method of Jorde and Ryman (2007; *N*_*e(JR)*_) with all possible combinations formed by subsequent and triennial samplings of the five yearly cohorts available. Both the original and the adjusted for overlapping generations estimates of *N*_*e(JR)*_ are given. The 95% confidence intervals of the original estimates are in brackets. Sampling sizes for each sample regime are also provided

Sample regime	Sample size	*N*_*e(JR)*_ estimates		Confidence intervals
		Original	Adjusted	
Subsequent cohorts				
From Cohort_2006_ to Cohort_2007_	74–136	6.0	13.4	(4.0;11.9)
From Cohort_2007_ to Cohort_2008_	136–211	14.4	32.1	(11.3;19.7)
From Cohort_2008_ to Cohort_2009_	211–225	7.0	15.6	(4.8;13.6)
From Cohort_2009_ to Cohort_2010_	225–134	14.8	33.0	(11.4;20.9)
Triennial sampling				
From Cohort_2006_ to Cohort_2008_	74–211	11.4	25.4	(8.0;19.4)
From Cohort_2007_ to Cohort_2009_	136–225	11.6	25.8	(7.0;32.3)
From Cohort_2008_ to Cohort_2010_	211–134	10.6	23.6	(7.8;16.4)

## Discussion

The Gochu Asturcelta pig breed offers a very particular scenario useful to illustrate the performance of single‐sample methods to estimate *N*_*e*_ in animal populations using molecular information. The breeding policy implemented by the breeders association allows to identify individuals that can be classified into discrete filial generations and, therefore, to compare the performance of different methods to estimate *N*_*e*_ under two different scenarios: generations overlap and discrete generations.

Genealogical estimates of effective size obtained using individual increase in inbreeding (*N*_*e*_ *F*_*i*_) and individual increase in coancestry (*N*_*e*_ *C*_*ij*_) kept consistency across reference populations (samples) and are in fully agreement with those recently reported by Menéndez et al. (2016a) for the most recent registered populations. Genealogical estimates are provided as a frame of reference for the understanding of the performance of the molecular‐based methods to estimate *N*_*e*_. Note that the genealogical methods applied correct for differences in pedigree depth and completeness of the individuals forming a reference population and, indirectly, account for the effects of mating policy, drift, overlap of generations, selection, and migration as a consequence of their reflection in the pedigree of each individual (Cervantes et al. 2008, 2009; Gutiérrez et al. 2008). Moreover, after the modification of the method suggested by Gutiérrez et al. (2009), and further applied for *N*_*e*_ *C*_*ij*_ by Cervantes et al. (2011a), *N*_*e*_ *F*_*i*_ accounts for the absence of self‐fertilization allowing to obtain useful estimates of *N*_*e*_ using pedigrees with three equivalents to complete generations on average. In the current analysis, the lower estimates of *N*_*e*_ *F*_*i*_ and *N*_*e*_ *C*_*ij*_ were assessed for the yearly Cohort (2006) with mean pedigree depth (*t *=* *2.7 ± 0.7) on the limit of estimability (Gutiérrez et al. 2009).

It is not surprising that molecular‐based estimates of *N*_*e*_ are higher than those obtained using genealogical data. Very recently, Silió et al. (2016), analyzing two experimental pig lines kept in herds closed for 24–28 generations and subject to a strict minimum coancestry mating policy, reported that molecular‐based estimates of *N*_*e*_ based on either inbreeding or coancestry tended to exceed their genealogical counterparts. Unlike pedigree information, which refers to a virtually infinite number of loci, criteria based on observed molecular polymorphism refer to a finite number of loci. In any case, sampling sizes and number of loci used here can be considered enough to obtain reliable estimates of effective populations size even if the expected *N*_*e*_ were moderate or large (Antao et al. 2010).

### Performance of the *N*_*e(LD)*_ method

Even when discrete filial generations are considered, estimates of *N*_*e(LD)*_ are at least threefold higher than the corresponding genealogical estimates (Table 2). However, estimates of *N*_*e(LD)*_ for filial generations F_3_ and F_4_ were lower and steadier than those obtained using yearly or biannual sampling. The linkage disequilibrium method relies on the fact that, in a system where gametes are randomly distributed among a small number of zygotes, there will be departures from expected genotype frequencies and departures from expected gametic frequencies, both of which can be used to estimate *N*_*e*_ (Hill 1981; Waples 1991). These assumptions only fit well to samples obtained from age‐structured populations. Moreover, in the case of overlapping generations, it is hard to assume that the available samples derive from a population with constant size. If population size changes, the “background” LD from previous generations that has not broken down by recombination between loci and new LD generated by reproduction of a finite number of individuals reflect different effective sizes and, therefore, estimate of *N*_*e*_ based on ${\hat{r}}^{2}$ can be biased upward or downward for a few generations (Waples 2005; Waples et al. 2014).

In any case, estimates of ${\hat{r}}^{2}$ obtained from molecular information in the Gochu Asturcelta pig breed can be biased upward even when discrete filial generations are considered. Demographic information allows to estimate ${\hat{r}}^{2}$ assuming selective neutrality and constant population size as ${\hat{r}}^{2}=\frac{1}{3H(N_{e},N_{b})}$, where *H*(*N*_*e*_ *,N*_*b*_) is the harmonic mean of *N*_*e*_ and *N*_*b*_ (see formula (5) in Waples et al. 2014). Demographic estimate of ${\hat{r}}^{2}$ would be here 0.00125 which underestimates the values of ${\hat{r}}^{2}$ obtained using molecular information whatever the sample considered (Table 2). Population studies ideally assume that LD is estimated using samples formed by unrelated individuals. This assumption is far from the pig population analyzed here and is not likely to occur in most livestock or natural animal populations therefore biasing upward the estimates of ${\hat{r}}^{2}$. Even though breeding policy of the Gochu Asturcelta population is under strict control, some hidden structuring, characterized by the ratio *N*_*e*_ *C*_*ij*_/*N*_*e*_ *F*_*i*_ (Cervantes et al. 2011a), has appeared probably due to an excessive use for reproduction of the descendants of two founders (Menéndez et al. 2016a).

Our results confirm that estimates of *N*_*e(LD)*_ obtained for filial generations F_3_ and F_4_ are more reliable than those assessed in scenarios with overlapping generations. Moreover, if no demographic adjustment is carried out, *N*_*e(LD)*_ estimates for yearly or biannual sampling schemes were terribly wrong (Table 2). Yearly samplings appeared clearly insufficient to obtain sound estimates of *N*_*e(LD)*_ *,* probably due to small sample size (Cohorts 2006 and 2010) or sampling bias (Cohort 2007; see Table 1). Estimates can be substantially biased at small sample sizes unless the true *N*_*e*_ was smaller than the sample size used to estimate it (England et al. 2006; Waples 2006). Although biased upward, the current results confirm that *N*_*e(LD)*_ estimates are more reliable when sampling span approaches a generation length (Waples et al. 2014). Such sampling span increases sample size, but also “homogenize” the actual number of breeders producing the sample across estimates. As theory suggests that *N*_*e(LD)*_ estimates are function of the harmonic mean of *N*_*e*_ and *N*_*b*_ (Waples et al. 2014), *N*_*e(LD)*_ should converge on true *N*_*e*_ when sampling span approaches the generation length.

### Performance of other molecular‐based methods to estimate *N*_*e*_

The Nomura (2008) coancestry‐based method gave lower estimates of *N*_*e*_ than *N*_*e(LD)*_ (Tables 2 and S3) and nearer to the “real” genealogical ones in the case of discrete filial generations. However, this may be due to the fact that *N*_*e(M)*_ is more likely related to the number of founders represented in the samples rather than population size. Caballero and Toro (2002) reported that 1/2*f* (being *f* the average molecular coancestry of the analyzed population) is actually the founder genome equivalents (*N*_*g*_). *N*_*g*_ is a key parameter to assess genetic losses due to drift which can be defined as the theoretically expected number of founders that would be required to provide the genetic diversity in the analyzed population if the founders were equally represented and had lost no alleles (Ballou and Lacy 1995). This definition is conceptually different to that of effective population size, the evolutionary analogous to census size, proposed by Wright (1931): the size of an idealized population which would give rise to the rate of inbreeding, or the rate of change in variance of gene frequencies, observed in the analyzed population. Even though the method by Nomura (2008) adjusts for the presence of alleles alike‐in‐state (Oliehoek et al. 2006), the main difference between his method and the Caballero and Toro's (2002) approach is that self‐coancestries (*s*_*i*_), the diagonals in the between‐individuals coancestry matrix (being $s_{i}=\frac{1+F_{i}}{2}$, where *F*_*i*_ is the homozygosity in a molecular context), are not included in the computations and, therefore, ${\hat{f}}_{}<f$ and *N*_*e(M)*_ > *N*_*g*_. Self‐coancestries have a major importance in computing *f*: the lower the sample size the higher the weight of self‐coancestries on *f* (Cervantes et al. 2011b). In any case, our results confirm the results by Miller et al. (2015) in bighorn sheep suggesting that ${\hat{f}}_{}$ tend to vary with ${\hat{r}}^{2}$. Therefore, both estimates of *N*_*e*_ (*N*_*e(M)*_ and *N*_*e(LD)*_) cannot be considered independent.

Results obtained using the Jorde and Ryman's (2007) approach illustrate that performance of temporal methods, when applied to data with overlapping generations, is highly dependent on sampling interval (Waples and Yokota 2007; Barker 2011) due to the particular age structure of the studied population. Here, *N*_*e(JR)*_ estimates obtained using subsequent yearly samplings did not accumulate sufficient drift signal, therefore giving inconsistent *N*_*e*_ estimates. In turn, too long separation among samples gives estimates of *N*_*e*_ highly biased upward (Table S4). Note that, in our example, genealogical separation between subsequent yearly samples is *t *≈* *0.5 while four‐year and five‐year sampling plans (Table S4) are separated by 1.5 and 2 equivalent to discrete generations (Table 2). The Jorde and Ryman's (2007) approach gave consistent estimates, comparable with adjusted *N*_*e(LD)*_ estimates obtained for biannual (generation interval) sampling, under a three‐year sampling plan. In our example, samples obtained under this sampling plan are separated by about *t *=* *1. This scenario is consistent with the performance of this method previously reported in horses (Goyache et al. 2011).

## Conclusions

The current results confirm the fact that performance of *N*_*e(LD)*_ can only be considered reliable in populations under generations overlapping when sampling span approaches a generation interval (Waples et al. 2014). Otherwise, sampling bias can affect the estimates of ${\hat{r}}^{2}$, probably due to unaccounted variation in molecular coancestry among samples. This may be particularly important in scenarios in which samples are not likely to be formed by unrelated individuals. Furthermore, *N*_*e(LD)*_ can only be considered useful if a correction of demographic bias is applied.

In such framework, even if no high variation of LD among yearly cohorts occur (Miller et al. 2015), the operational advantage of using single‐sample methods to obtain molecular‐based estimates of *N*_*e*_ is not clear: while two‐sample methods may need a sample span exceeding a generation interval, single‐sample methods (namely *N*_*e(LD)*_) will need a representative sampling in each of the yearly cohorts included in that interval. These concerns particularly apply to natural and domestic populations with large generation intervals. As an example, it is worth mentioning that in domestic horses, generation interval usually exceeds 10 years (Cervantes et al. 2009). In such scenario, it is hard to assume that available samples are representative of a complete generation interval period (Corbin et al. 2010, 2012).

The current study has been performed using LD between unlinked loci. The availability of high‐density SNP Chips offers the opportunity of estimating *N*_*e(LD)*_ using LD between linked loci, therefore improving the performance of the method. However, the concerns about sampling span described above still apply. Actually, high‐density SNP Chips have been used to ascertain the variation of *N*_*e*_ over time, expressed as generations in the past (Corbin et al. 2010, 2012; Flury et al. 2010). Even though some of these studies use complex models accounting for sources of variation such as sample size, mutation, phasing, or recombination rate together with thousands of linked SNP data (Corbin et al. 2012; Barbato et al. 2015), *N*_*e(LD)*_ estimates at a given point of time are always function of both ${\hat{r}}^{2}$ and between‐SNPs distance in Morgans (*c*). As fitting *c* is usually arbitrary, historical estimates of *N*_*e*_ mainly depend on ${\hat{r}}^{2}$ which, in turn, depends on sampling and demographic structure of the studied population.

Overall, the empirical evidence given in the current study confirms that estimates of *N*_*e*_ obtained using methods based in molecular information should be interpreted with caution (Barker 2011; Goyache et al. 2011; Putman and Carbone 2014).

## Supporting information


**Table S1**. Life table used to calculate the ratio between effective number of breeders (*N*_*b*_) and effective population size (*N*_*e*_) using demographic data, as proposed by Waples et al. (2014, see references section).
**Table S2**. Life table used to calculate the correction factor (*C*) for overlapping generations proposed by Jorde and Ryman (1995, 1996, see references section).
**Table S3**. Number of individuals (N) involved and estimates of effective size for three‐years sampling in the Gochu Asturcelta pig breed population computed via molecular‐based methods (linkage disequilibrium, *N*_*e(LD)*_, and molecular coancestry, *N*_*e(M)*_) and pedigree information (individual increase in inbreeding, *N*_*e*_ *F*_*i*_, and individual increase in coancestry, *N*_*e*_ *C*_*ij*_).
**Table S4**. Estimates of *N*_*e*_ obtained in the Gochu Asturcelta pig population using the temporal method of Jorde and Ryman (2007; *N*_*e(JR)*_) with all possible four‐year and five‐year sampling plans formed with combinations of the five yearly cohorts available.
