# Supplementary material for: Assessing performance of single‐sample molecular genetic methods to estimate effective population size: empirical evidence from the endangered Gochu Asturcelta pig breed
Source: Ecol Evol. 2016 Jun 23;6(14):4971–80. doi: 10.1002/ece3.2240 (PMC4979721; doi:10.1002/ece3.2240)
Supplement: Supplementary file 1 — Table S1. Life table used to calculate the ratio between effective number of breeders (N b) and effective population size (N e) using demographic data, as proposed by Waples et al. (2014, see references section). Table S2. Life table used to calculate the correction factor (C) for overlapping generations proposed by Jorde and Ryman (1995, 1996, see references section). Table S3. Number of individuals (N) involved and estimates of effective size for three‐years sampling in the Gochu Asturcelta pig breed population computed via molecular‐based methods (linkage disequilibrium, N e(LD), and molecular coancestry, N e(M)) and pedigree information (individual increase in inbreeding, N e F i, and individual increase in coancestry, N e C ij). Table S4. Estimates of N e obtained in the Gochu Asturcelta pig population using the temporal method of Jorde and Ryman (2007; N e(JR)) with all possible four‐year and five‐year sampling plans formed with combinations of the five yearly cohorts available. [file ECE3-6-4971-s001.doc]

**Supplementary** **Table S1.** Life table used to calculate the ratio between effective number of breeders (*N_b_*) and effective population size (*N_e_*) using demographic data, as proposed by Waples et al (2014, see references section). Age-specific survival rates (*s_x_*; i.e. probability of surviving from age *x* to age *x* + 1) and birth rates (*b_x_*; i.e. mean number of offspring in one time period produced by a parent of age *x*) were calculated separately for males and females directly from the Gochu Asturcelta pedigrees limiting the age of the parents to 5 years old. Computations were carried out assuming the harmonic mean of the yearly cohorts registered from 2006 to 2010 (133) as parameter N_1_ (total number of offspring per time period that survive to age 1) and no different Poisson variance in reproductive success among same-sex and same-age individuals.

|  | *males* | | *females* | |
| --- | --- | --- | --- | --- |
| Age class (*i*) | *l_i_* | *b_i_* | *l_i_* | *b_i_* |
| 1^a^ | 0.461 | 6.6 | 0.389 | 6.6 |
| 2 | 0.360 | 6.6 | 0.392 | 7.7 |
| 3 | 0.133 | 5.9 | 0.169 | 6.6 |
| 4 | 0.026 | 5.8 | 0.043 | 4.2 |
| 5 | 0.020 | 4.3 | 0.008 | 6.2 |

^a^in years

**Supplementary** **Table S2.** Life table used to calculate the correction factor (*C*) for overlapping generations proposed by Jorde and Ryman (1995, 1996, see references section). Age-specific survival rates (*l_i_*) and birth rates at each age class *i* (gametic contribution; *b_i_*) are listed. Parameters *l_i_* and *b_i_* were estimated directly from the Gochu Asturcelta pedigrees limiting the age of the parents to 5 years old. Note that if data do not result in a constant population size, the model by Jorde and Ryman (1995, 1996) adjusts all *b_i_*’s to yield Sum *l_i_** *b_i_* = 1.

| Age class (*i*) | *l_i_* | *b_i_* |
| --- | --- | --- |
| 1^a^ | 0.4251 | 1.97 |
| 2 | 0.3755 | 1.90 |
| 3 | 0.1510 | 1.49 |
| 4 | 0.0344 | 5.56 |
| 6 | 0.0141 | 2.31 |

^a^in years

**Supplementary Table S3.** Number of individuals (N) involved and estimates of effective size for three-years sampling in the Gochu Asturcelta pig breed population computed via molecular-based methods (linkage disequilibrium, *N_e(LD)_*, and molecular coancestry, *N_e(M)_*) and pedigree information (individual increase in inbreeding, *N_e_F_i_*, and individual increase in coancestry, *N_e_C_ij_*). In brackets, confidence intervals of the estimates on 95% (molecular-based methods) or standard errors of the estimates (genealogical methods) are provided. Additionally, the estimated correlation ($\hat{r}^{2}$) and molecular coancestry ($\hat{f}$) among alleles are given for the molecular-based methods and mean inbreeding (*F*), mean equivalent to discrete generations (*t*) and average individual increase in inbreeding ($\overline{\Delta F_{i}}$) are provided for pedigree data.

|  | Molecular estimates | | | | | Genealogical estimates | | | | | |  |
| --- | --- | --- | --- | --- | --- | --- | --- | --- | --- | --- | --- | --- |
| Three-annual Sampling | N^†^ | $\hat{r}^{2}$ | *N_e(LD)_*^‡^ | $\hat{f}$ | *N_e(M)_* | N^a^ | *F* | *t* | $\overline{\Delta F_{i}}$ | *N_e_F_i_* | *N_e_C_ij_* | $\frac{N_{e}C_{ij\text{ }}}{N_{e}F_{i}}$ |
| Sampling_2006-2008_ | 421 | 0.00239 | 23.9^§^ [35.8^††^ (29.4; 43.2)] | 0.04412 | 11.3 (5.0; 20.1) | 881 | 0.21 ± 0.08 | 3.5 ± 0.6 | 0.09 ± 0.04 | 5.6 ± 1.1 | 6.3 ± 0.5 | 1.13 |
| Sampling_2007-2009_ | 572 | 0.00176 | 24.6^§^ [36.9^††^ (30.7; 44.0)] | 0.04279 | 11.7 (6.9; 17.7) | 1522 | 0.22 ± 0.06 | 3.9 ± 0.6 | 0.08 ± 0.03 | 6.0 ± 0.8 | 7.1 ± 0.4 | 1.18 |
| Sampling_2008-2010_ | 570 | 0.00176 | 22.6^§^ [33.9^††^ (28.9; 39.3)] | 0.03746 | 13.3 (7.2; 21.4) | 1930 | 0.23 ± 0.06 | 4.2 ± 0.6 | 0.08 ± 0.02 | 6.3 ± 0.7 | 7.9 ± 0.4 | 1.25 |

^†^Number of individuals involved in the estimates

^‡^Values obtained removing alleles with frequencies (*P_crit_*) lower than 0.05

^§^Estimates of effective size after correction for bias due to age structure

^††^Original estimates of effective size and confidence intervals

**Table S4.** Estimates of *N_e_* obtained in the Gochu Asturcelta pig population using the temporal method of Jorde and Ryman (2007; *N_e(JR)_*) with all possible four-year and five-year sampling plans formed with combinations of the five yearly cohorts available. Both the original and the adjusted for overlapping generations estimates of *N_e(JR)_* are given. The 95% confidence intervals of the original estimates are in brackets. Sampling sizes for each sample regime are also provided.

| Sample | Sample | *N_e(JR)_* estimates | | Confidence |
| --- | --- | --- | --- | --- |
| regime | size | original | adjusted | Intervals |
| Four-year sampling |  |  |  |  |
| From Cohort_2006_ to Cohort_2009_ | 74-225 | 18.2 | 40.5 | (11.0;52.5) |
| From Cohort_2007_ to Cohort_2010_ | 136-134 | 18.1 | 40.3 | (10.4;69.1) |
| Five-year sampling |  |  |  |  |
| From Cohort_2006_ to Cohort_2010_ | 74-134 | 25.8 | 57.5 | (14.8;101.3) |
